# Plasma Testosterone Levels and Atherosclerotic Plaque Gene Expression in Men With Advanced Atherosclerosis

**DOI:** 10.3389/fcvm.2021.693351

**Published:** 2021-06-14

**Authors:** Floor Groepenhoff, Ernest Diez Benavente, Arjan Boltjes, Nathalie Timmerman, Farahnaz Waissi, Robin J. G. Hartman, N. C. Onland-Moret, Gerard Pasterkamp, Hester Den Ruijter

**Affiliations:** ^1^Laboratory of Experimental Cardiology, University Medical Center Utrecht, Utrecht University, Utrecht, Netherlands; ^2^Central Diagnostic Laboratory, University Medical Center Utrecht, Utrecht University, Utrecht, Netherlands; ^3^Division of Surgical Specialties, Department of Vascular Surgery, University Medical Centre Utrecht, Utrecht University, Utrecht, Netherlands; ^4^Julius Center for Health Sciences and Primary Care, University Medical Center Utrecht, Utrecht University, Utrecht, Netherlands

**Keywords:** testosterone, atherosclerosis, RNA-expression, transcriptome (RNA-seq), men

## Abstract

**Aims:** Low plasma testosterone levels have been shown to predict worse outcome in men with severe atherosclerotic disease. We hypothesized that a low plasma testosterone level affects disease risk through changes in gene expression in atherosclerotic plaques. Therefore, we studied plasma testosterone levels in relation to gene expression levels in atherosclerotic plaque tissue of men with advanced atherosclerotic disease.

**Methods:** Plasma testosterone levels were measured in 203 men undergoing carotid endarterectomy. The corresponding atherosclerotic plaque tissue was used for RNA sequencing. First, we assessed how often the androgen receptor gene was expressed in the plaque. Second, correlations between plasma testosterone levels and pre-selected testosterone-sensitive genes were assessed. Finally, differences within the RNA expression profile of the plaque as a whole, characterized into gene regulatory networks and at individual gene level were assessed in relation to testosterone levels.

**Results:** Testosterone plasma levels were low with a median of 11.6 nmol/L (IQR: 8.6–13.8). RNA-seq of the plaque resulted in reliable expression data for 18,850 genes to be analyzed. Within the RNA seq data, the androgen-receptor gene was expressed in 189 out of 203 (93%) atherosclerotic plaques of men undergoing carotid endarterectomy. The androgen receptor gene expression was not associated with testosterone plasma levels. There were no significant differences in gene expression of atherosclerotic plaques between different endogenous testosterone levels. This remained true for known testosterone-sensitive genes, the complete transcriptomic profile, male-specific gene co-expression modules as well as for individual genes.

**Conclusion:** In men with severe atherosclerotic disease the androgen receptor is highly expressed in plaque tissue. However, plasma testosterone levels were neither associated with pre-selected testosterone sensitive genes, gene expression profiles nor gene regulatory networks in late-stage atherosclerotic plaques. The effect of testosterone on gene expression of the late-stage atherosclerotic plaque appears limited, suggesting that alternate mechanisms explain its effect on clinical outcomes.

## Introduction

Testosterone is the most important sex hormone in men. The decline in testosterone levels with age in men has been linked to increased prevalence of (coronary) artery disease and cardiovascular events (1). In men, higher plasma testosterone levels have shown to protect against cardiovascular disease (2, 3). Therefore, supplementation of testosterone in men with low testosterone levels was considered to be beneficial. However, treatment of low testosterone levels in men have resulted in ambiguous results (4), stressing the need to unravel the underlying mechanisms responsible for the detrimental effect of low endogenous testosterone in men.

Atherosclerosis underlies the majority of cardiovascular disease (5). The composition of the plaque is an important indicator of cardiovascular events (6). Animal studies have shown a beneficial effect of testosterone on atherosclerotic plaque formation, suspectedly *via* its anti-inflammatory properties (7, 8). In humans, even though the relationship between endogenous testosterone levels and cardiovascular disease has extensively been studied (3), the role of testosterone in the development and composition of the plaque is still unknown.

Testosterone is the main endogenous androgen (9) and is involved in many physiological processes, among which the function of the vascular endothelium. For example, by binding to the nuclear androgen receptor, testosterone can alter downstream gene expression resulting in vasodilatation. To exert its regulatory effect, testosterone needs to bind to the androgen receptor (10), this receptor is nuclear and the predominant mediator of the effects of endogenous testosterone. For example, androgen receptor ablation in the vascular smooth muscle cell inhibits vascular calcification (11). The androgen receptor regulates downstream effects of testosterone as a transcription factor and it is expressed in many tissues, including the vascular endothelium. However, besides genomic actions of testosterone on the vasculature, it is thought to also have non-genomic vasoactive effects which might be exerted through interaction with proteins, receptors, or ion channels nested in the plasma membrane (9).

As we previously showed that plasma testosterone was a strong predictor of a secondary stroke in our cohort of men undergoing carotid endarterectomy (12), we hypothesized that the mechanism by which low testosterone levels affect cardiovascular risk is through the atherosclerotic plaque. To test this hypothesis, we studied if plasma testosterone associates with plaque tissue gene expression. We used several approaches including analyses of complete transcriptomic profiles as well as gene co-expression networks as these are able to capture relatively subtle changes in overall expression within tissues (13). We also studied if pre-selected testosterone-sensitive genes and receptors were associated with plasma testosterone levels.

## Methods

### Study Population

We analyzed transcriptional activity by RNA-sequencing of plaques from men included in the Athero-Express Biobank Study (14) of whom testosterone level and RNA-sequencing data were available (*n* = 203). Details of the Athero-Express study protocol have been described previously (12, 15). In short, patients undergoing endarterectomy of the carotid artery in two Dutch tertiary referral centers between 2002 and 2015 were included in this study. Study procedures comprise of a baseline blood withdrawal, an extensive questionnaire filled in by the participants verified against medical records, and collection of carotid arterial plaque material during surgery. All patients provided written informed consent before surgery, the study was approved by the Local Medical Ethical Committee and conducted according to the Declaration of Helsinki (16).

### Sex Steroid Measurements

Serum testosterone was measured by immunoassay on an ARCHITECT ci8200 system (Abbott Diagnostics, Abbott laboratories, USA) (12).

### Histology

As described previously (12, 14, 17), the atherosclerotic plaque was processed directly after surgery and (immune-) histochemical staining was routinely performed on the culprit lesion (segment with the highest plaque burden) for identification of macrophages (CD 68), calcification (haematoxylin-eosin (HE)), smooth muscle cells (alfa actin), collagen [Picro Sirius red (PSR)], intra-plaque hemorrhage (HE, Elastin von Gieson staining), vessel density (CD34) and fat (PSR, HE).

### Bulk RNA Sequencing

As the culprit lesion is used for plaque histology following the standardized Athero-Express protocol (14), the adjacent plaque segments were used for RNA sequencing. To measure bulk RNA expression in the plaques total RNA was isolated according to the manufacturers protocol after processing of the plaque segments using ceramic beads and tissue homogenizer (Precellys, Bertin intruments, Montigny-le-Bretonneux) with use of TriPure (Sigma Aldrich). After precipitating RNA in the aqueous phase with propanolol, RNA was washed with 75% ethanol and either used immediately after an additional washing step with 75% ethanol or stored in 75% ethanol for later use. Subsequently, library preparation was performed as described before (18–20). Ethanol was removed and the pellet air-dried. Then, primer mix (5 ng primer per reaction) was added to initiate primer annealing at 65 degrees Celsius for 5 min. Subsequently, reverse transcription (RT) was executed. Subsequent reverse-transcription RT reaction; first strand reaction for 1 h at 42°C, heat inactivated for 10 min at 70°C, second strand reaction for 2 h at 16°C, and then put on ice until proceeding to sample pooling.

This initial RT reaction used the following primer design: an anchored polyT, a unique 6 bp barcode, a unique molecular identifier (UMI) of 6 bp, the 5' Illumina adapter and a T7 promoter, as described (18–20). Each sample now contained its own unique barcode making it possible to pool together cDNA samples at 7 samples per pool. Complementary DNA (cDNA) was cleaned using AMPure XP beads (Beckman Coulter), washed with 80% ethanol, and resuspended in water before proceeding to the *in vitro* transcription (IVT) reaction (AM1334; Thermo-Fisher) incubated at 37°C for 13 h. Next, Exo-SAP (Affymetrix, Thermo-Fisher) was used to remove primers, upon which amplified RNA (aRNA) was fragmented, cleaned with RNAClean XP (Beckman-Coulter), washed with 70% ethanol, air-dried, and resuspended in water. RNA yield and quality in the suspension were checked by Bioanalyzer (Agilent) after removal of the beads with use of a magnetic stand. By performing an RT reaction using SuperScript II reverse transcriptase (Invitrogen/Thermo-Fisher) according to the protocol of the manufacturer cDNA library construction was initiated. Next, PCR amplification was performed as described previously (18–20). PCR products were cleaned twice using AMPure XP beads (Beckman Coulter). Qubit fluorometric quantification (Thermo-Fisher) and Bioanalyzer (Agilent) were used to checked Library cDNA yield and quality. Illumina Nextseq500 platform was used to sequence the libraries; paired end, 2 × 75 bp. After sequencing, retrieved fastq files were de-barcoded and split into forward and reverse reads. From there, the reads were mapped using Burrows-Wheel aligner (BWA37) version 0.7.17–r1188, calling “bwa aln” with settings -B 6 -q 0 -n 0.00 -k 2 -l 200 -t 6 for R1 and -B 0 -q 0 -n 0.04 -k 2 -l 200 -t 6 for R2, “bwa sampe” with settings -n 100 -N 100, and a cDNA reference (assembly hg19, Ensembl release 84). Read and UMI counts were acquired from SAM files with use of custom perl code and collected into count matrices. Further analyses were performed using R (21) version 3.6.2 and later and its IDE Rstudio (22) version 1.2 and later. Genes were annotated with Ensembl ID's, basic quality control was performed [filtering out samples with low gene numbers (<10,000 genes) and read counts (<18,000 reads)].

### Statistical Analyses

Counts, metadata and clinical data were combined into a SummarizedExperiment (23) object. Counts were pre-filtered, normalized and transformed making use of the variance stabilization transformation function (vst) in DESeq2 (24), resulting in transformed data of *n* = 203 on a log2-scale, normalized for library size (24). These were used to visualize gene expression, to assess correlations between gene expression and androgen receptor expression and plasma testosterone levels, to construct correlation heatmaps, correlation scatter plots, and Manhattan plots, furthermore we used these to calculate differentially expressed genes between high and low testosterone tertiles. Correlation estimates and *p*-values were calculated using Spearman's rank correlation. Correlations between the expression levels of genes and testosterone level were explored using scatterplots. Heatmaps were drawn using Pheatmap from the Pheatmap package (25), applying hierarchical clustering based on correlation estimates with standard settings: complete linkage and Euclidean distance.

### Gene Regulatory Networks Analysis

The software package WGCNA (13) was used to generate modules of co-expression genes on the 486 available men RNAseq samples. After excluding all the ribosomal genes and included only the protein-coding genes with annotated HGCN names, a set of 12,765 protein-coding genes which passed quality control (average >1 count per sample) was used for module generation. The raw read counts were corrected for UMI sampling [corrected_count = −096^*^(ln (1–(raw_count/4096)))], normalized by sample sequencing depth and log-transformed. A signed network was constructed using the robust “bicor” correlation measure. To determine the exponent used for the adjacency matrix construction, soft thresholding analysis was performed with the WGCNA package for powers ranging from 2 to 30. The cut-off for assuming scale-free topology was set at an R-squared of 0.8, while having a median connectivity of lower than 100.The chosen lowest complying power was 26. The network was constructed by first generating an adjacency matrix, which was transformed into a topological overlay matrix (13). Modules were detected by clustering the average distance of the dissimilarity matrix (defined as 1- topological overlay matrix) and cutting the subsequent dendrogram by using the cutreeDynamic function (deepSplit = 2, minClusterSize = 20). Module eigengenes were calculated by taking the first principal component of gene expression in that module. Module eigengene values were selected for the 203 samples with testosterone measurements available. The module eigengenes were correlated to clinical traits by Pearson correlation, with a Student asymptomatic *p*-value test for significance.

### Data and Scripts Availability

Data and scripts are available upon reasonable request.

## Results

### Baseline Characteristics

We analyzed data of 203 men with a mean age of 68 years old. Most men presented with a transient ischemic attack (42%) or stroke (23%). Median testosterone level was 11.6 nmol/L (IQR: 8.6–13.8). The participants of the study population were allocated to tertiles based on testosterone levels: high (median 16.5, IQR: 14.9–18.9 nmol/L), medium (11.6, IQR: 10.6–12.7 nmol/L), and low (7.6, IQR: 8.6–13.8 nmol/L). Patient characteristics of our study population stratified by testosterone tertiles are presented in Table 1. Age, body mass index and renal function did not differ between the tertiles. Hypercholesterolemia seemed more prevalent in the low and medium testosterone level group (73 and 72%, respectively) as compared to the group with the highest testosterone levels (60%), yet this difference was not statistically significant. Hypertension was most prevalent in individuals with the lowest testosterone levels [83% in the low, 69% in the medium and 59% in the highest testosterone tertile, respectively (*p* < 0.05)]. In all groups, a transient ischemic attack was the most common clinical presentation and there was a balanced distribution of plaque phenotypes based on histology with similar prevalence of atheromatous, fibro-atheromatous, and fibrous plaques (17).

**Table 1:** Baseline characteristics by testosterone tertiles of men undergoing carotid endarterectomy.

	**Overall**	**Low**	**Medium**	**High**
*n*	203	68	68	67
Testosterone nmol/mL [median (IQR)]	11.6 (8.6–13.8)	7.6 (5.7–8.6)	11.6 (10.6–12.7)	16.5 (14.9–18.9)
Age [years; mean (SD)]	68 (8.8)	69 (9.6)	69 (7.5)	68 (9.2)
Body mass index [mean (SD)]	26.6 (3.6)	27.0 (3.9)	26.8 (3.64)	25.8 (3.3)
Renal function [GFR; mean (SD)]	75 (22)	74 (26)	73 (18)	79 (20)
Total Cholesterol [mean (SD)]	4.0 (1.1)	3.7 (1.0)	4.0 (1.2)	4.4 (1.1)
Triglycerides [mean (SD)]	1.6 (0.8)	1.7 (0.9)	1.7 (0.7)	1.5 (0.8)
LDL [mean (SD)]	2.3 (0.9)	2.1 (0.8)	2.3 (0.9)	2.5 (0.9)
HDL [mean (SD)]	1.0 (0.3)	0.9 (0.3)	1.0 (0.3)	1.1 (0.3)
Current smoker = yes, *n* (%)	70 (35)	23 (34)	26 (38)	21 (31)
Diabetes Mellitus = yes, *n* (%)	37 (18)	19 (28)	13 (19)	5 (8)
Hypercholesterolemia = yes, *n* (%)	127 (68)	45 (73)	46 (72)	36 (60)
Hypertension = yes, *n* (%)	135 (69)	52 (79)	46 (69)	37 (59)
History of coronary artery disease = yes, *n* (%)	65 (32)	22 (32)	24 (35)	19 (28)
History of stroke = yes, *n* (%)	54 (27)	22 (32)	16 (24)	16 (24)
History of peripheral artery disease = yes, *n* (%)	44 (22)	19 (28)	15 (22)	10 (15)
Symptoms group, *n* (%)				
Asymptomatic	38 (19)	11 (17)	16 (24)	11 (17)
Ocular	31 (16)	11 (17)	10 (15)	10 (15)
Stroke	46 (23)	18 (28)	13 (19)	15 (23)
Transient ischemic attack	84 (42)	25 (39)	29 (43)	30 (46)
Plaque phenotype, *n* (%)				
Atheromatous	63 (32)	23 (34)	18 (27)	22 (33)
Fibro-atheromatous	67 (34)	22 (33)	22 (33)	23 (35)
Fibrous	70 (35)	22 (33)	27 (40)	21 (32)
Statins or lipid lowering medication = yes, *n* (%)	158 (78)	54 (79)	53 (78)	51 (76)
Antiplatelet medication = yes, *n* (%)	186 (92)	60 (88)	63 (93)	63 (94)

### Androgen Receptor Expression Within the Atherosclerotic Plaque

**Figure 1:**
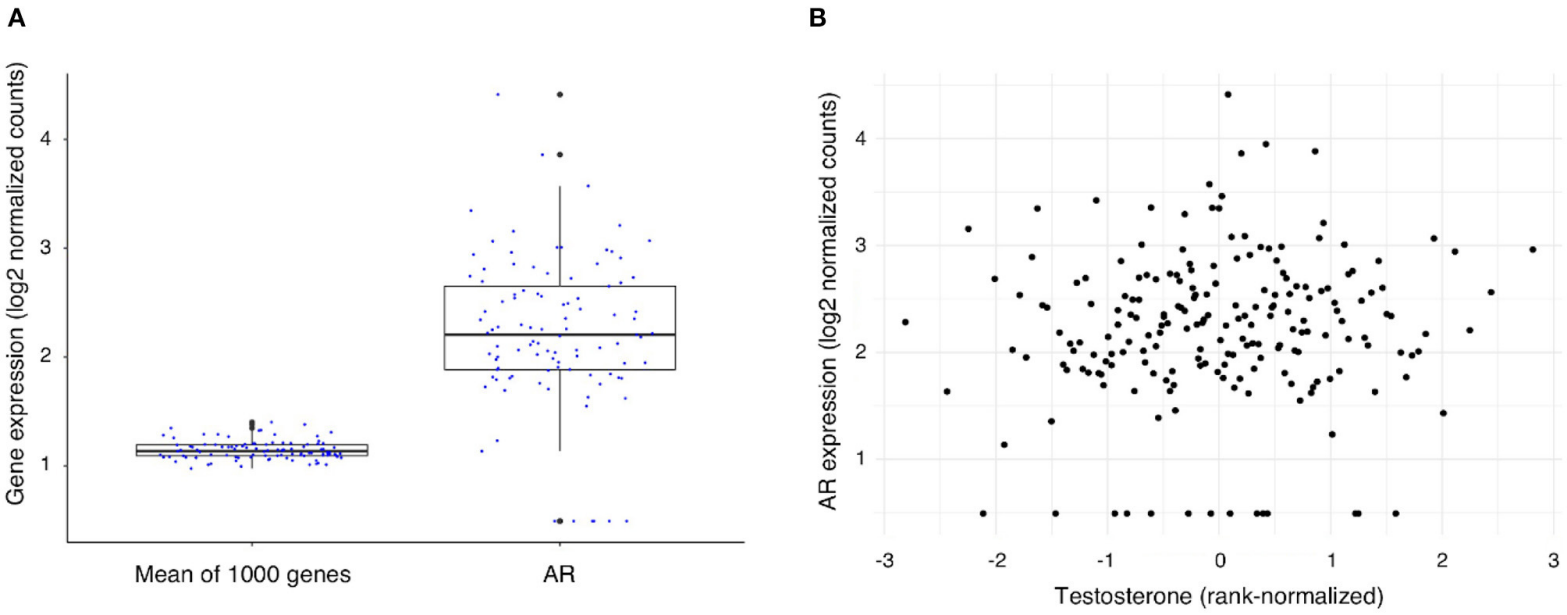
Expression of the Androgen Receptor (AR) gene and its association with testosterone levels in men undergoing carotid endarterectomy. **(A)** AR gene expression levels as compared with the mean expression level of 1,000 randomly selected genes. **(B)** Scatterplot of testosterone level (rank-normalized) and AR gene expression in plaque.

To exert its function, testosterone needs to bind to the androgen receptor (AR) (10) which mediates almost all the known genomic effects of testosterone. To assess the level of AR expression within the atherosclerotic plaque we compared its expression to the mean expression of 1,000 randomly selected genes (Figure 1A), the mean expression of the AR gene was 2.2, SD: 0.7 vs. 1.1, SD: 0.1 for the average of 1,000 genes (log transformed counts). We found that the AR gene is expressed within the atherosclerotic plaques of 93% (189 of 203 patients) in our male cohort (count > 1). AR expression levels were not significantly correlated to testosterone levels in serum of the same patients (Figure 1B) (Spearman rank correlation (*rho*): 0.08, unadjusted *p*-value = 0.25). In addition, we explored the expression of other nuclear receptors in relationship to the AR gene, several receptors presented with comparable expression levels with NR6A, HNF4A, and THRB presenting the highest expression in this cohort (Supplementary Figure 1).

### Testosterone-Sensitive Genes and Plasma Testosterone Levels

**Figure 2:**
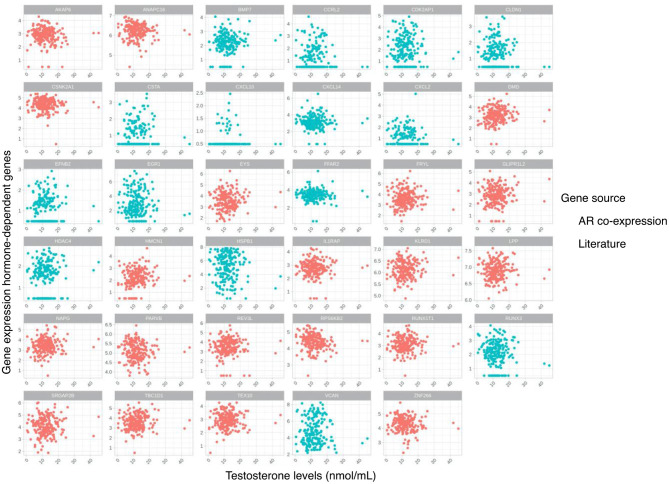
Scatterplots for testosterone levels and gene expression of selected hormone-dependent genes based on literature and gene co-expression with androgen receptor (AR) gene in the atherosclerotic plaque.

A list of testosterone-sensitive genes was selected based on literature (26). In addition, an unbiased list of genes was selected based on significant correlation (after Bonferroni adjustment) of their gene expression with expression of the AR gene (Supplementary Figure 2, Supplementary Table 1), 12 out of these 20 genes (60%) were identified as being targets of the AR gene as a transcription factor (27). The combination of these two lists of candidate testosterone-sensitive genes was used, and an association of their expression with testosterone levels within the plaque was further evaluated (Table 2). Correlation analysis of the testosterone-sensitive genes' expression (Table 2) and continuous testosterone levels showed no significant correlations (Figure 2). Clustering of individuals using hierarchical clustering and based on sample distances restricted to the testosterone-sensitive gene subset did not show an overlap with the established testosterone-level tertiles (Supplementary Figure 3).

**Table 2:** Selected genes for testosterone-sensitivity.

**Selected based on literature (26)**	**Selected based on correlation with AR gene expression**
CXCL10, EGR1, VCAN, BMP7, FGF3, EFNB2, CLDN1, CXCL2, CSTA, CDK2AP1, HDAC4	NAPG, SRGAP2B, PARVB, DMD, TBC1D1, FRYL, CSNK2A1, GLIPR1L2, HMCN1, KLRD1, REV3L, TEX10, EYS, RPS6KB2, RUNX1T1, IL1RAP, AKAP6, ZNF266, LPP, ANAPC16

*AR, androgen receptor*.

### Plaque Transcriptional States Do Not Correlate With Testosterone Levels

**Figure 3:**
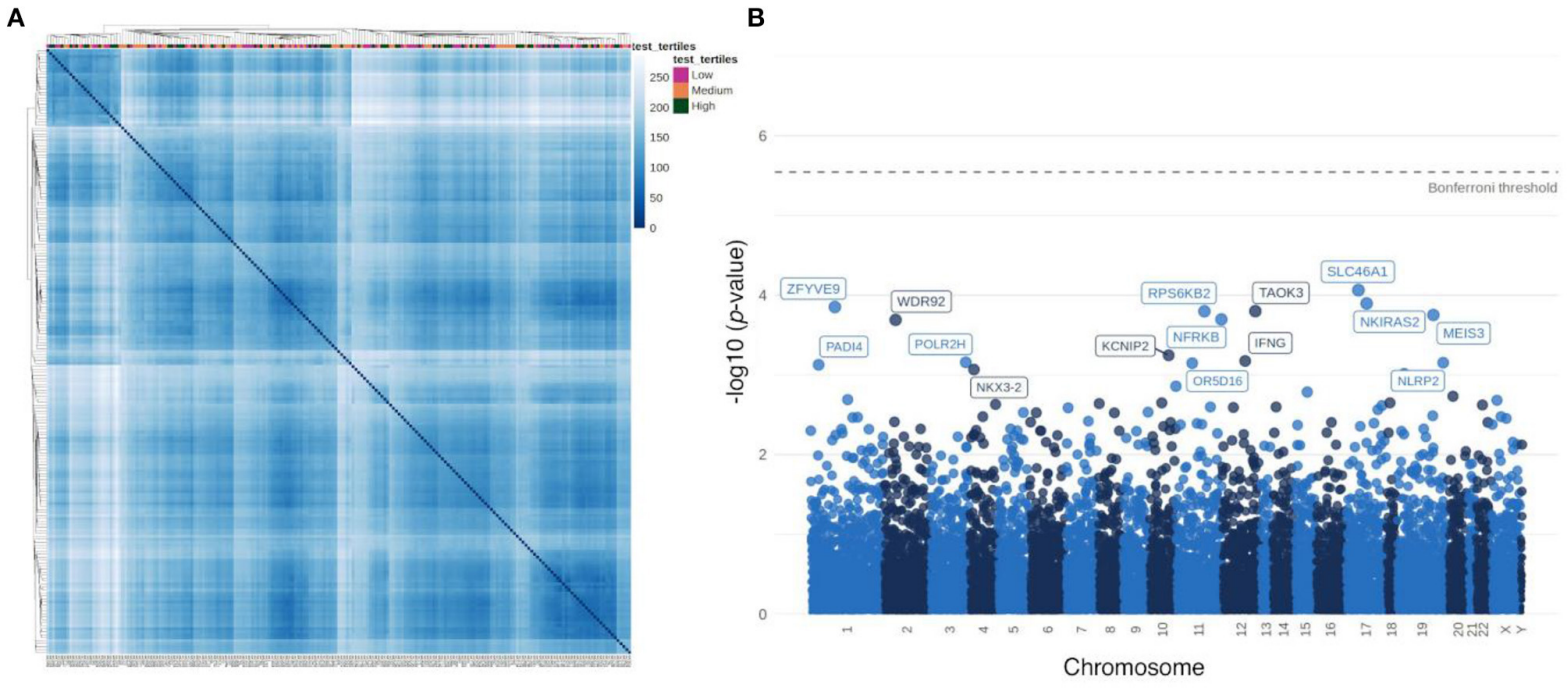
Association of testosterone levels with transcriptome signatures in male plaque tissue. **(A)** Heatmap based on sample-to-sample distances colored by continuous testosterone levels. **(B)** Manhattan plot of associations between testosterone levels and individual gene expression.

Hierarchical clustering based on sample distances did not highlight a group of patients who are similar with respect to their specific testosterone levels (Figure 3A). In order to identify individual genes associated with testosterone levels, correlations between gene expression from each gene with testosterone levels were tested. No gene was statistically significantly correlated with testosterone (after Bonferroni correction). The top 15 genes nominally correlated with testosterone levels are labeled in a Manhattan plot in Figure 3B and presented in Supplementary Table 2. We also performed differential expressed analysis between high and low testosterone levels. Yet, there were no differentially expressed genes (data not shown).

### No Evidence of an Effect of Testosterone Levels on Male-Specific Gene Co-expression Modules

**Figure 4:**
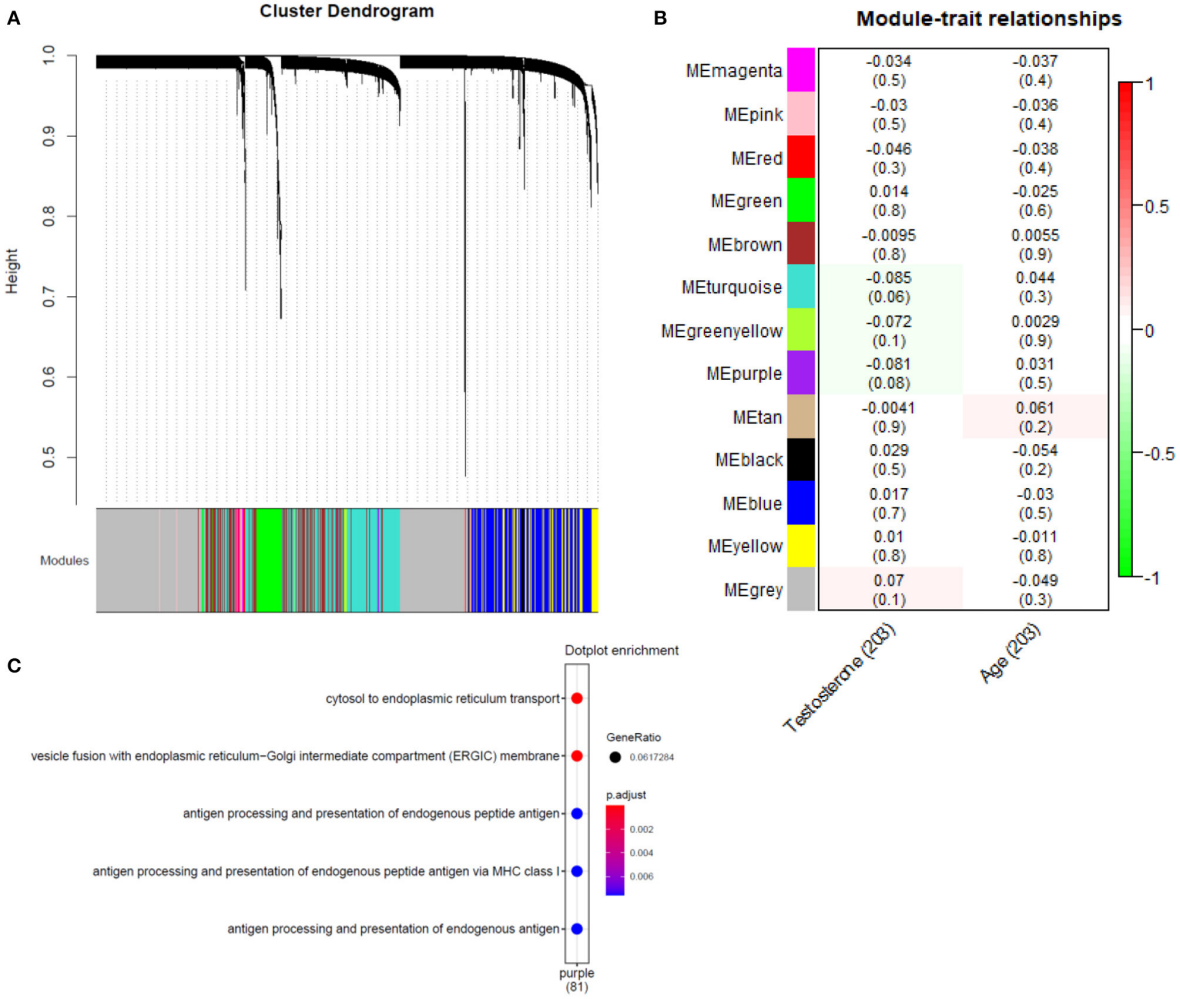
Association analysis of gene co-expression networks with testosterone levels. **(A)** Hierarchical cluster tree of 12,756 genes, height (y-axis) is determined using the average gene linkage from the topological overlap measure, which incorporates information about both co-expression and connectivity in the network. A total of 14 modules were assigned based on the dynamic hybrid branch cutting method and are shown along the x-axis in different colors. Genes in the same module have higher co-expression and present higher connection in the network. **(B)** Correlation of module eigengenes (a composite measure of the combined expression of the genes from each module) with testosterone levels and age of the participants, *p*-values for the correlation are in parenthesis. **(C)** Gene enrichment GO terms obtained for the purple module, nominally associated with testosterone levels (no significant enrichment was found for turquoise module).

Gene co-expression modules were constructed following previously described methods (28, 29) (Supplementary Figure 4), identifying 14 modules (Figure 4A) in the bulk plaque RNA-seq data. Eigen genes were calculated for each individual module, representing a proxy for the combined expression of the genes that belong to each module. Using this composite measure of module expression, we correlated each module with the rank-normalized testosterone levels in the 203 patients with data available. Two modules (purple and turquoise) were identified with nominally significant negative correlations (*p*-value < 0.1) (Figure 4B). However, no significant associations remained after adjusting the *p*-values using Holm method for false discovery rate (30). To understand the biological relevance of such modules, gene enrichment analysis was performed and the modules that were nominally associated were pointing to endoplasmic reticulum processes (Figure 4C).

## Discussion

By using statistical and system biology approaches, we show that gene expression of the plaque tissue of men with severe atherosclerosis is unrelated to plasma testosterone levels, despite a high relative expression of the androgen receptor.

Testosterone level itself was not correlated with the overall transcriptional state of the atherosclerotic plaques in our study and did not significantly influence gene expression at individual gene level. Furthermore, dissecting the gene expression profiles into gene co-expression modules, which are known to reveal subtle differences in tissue gene expression (29) also showed no effect of testosterone on gene regulatory networks. Also, known testosterone-sensitive (26) genes as well as genes that were correlated to the androgen receptor expression were not correlated to plasma testosterone levels.

Several factors might explain our unexpected results. First of all, levels in our study were overall low as our population consists mainly of older men. It may well be that testosterone does affect plaque expression, and thereby affects atherosclerosis, but rather at an earlier age when testosterone levels are higher. Despite the low levels, we did observe a strong relation between sex-hormone levels and secondary outcomes (12) in this cohort. Low testosterone to estradiol predicted future major cardiovascular events (hazard ratio (HR) 1.67; 95% CI: 1.02–2.76, an effect even stronger in obese men (HR 2.42; 95% CI: 1.09–5.38), This coincided with an unfavorable inflammatory pattern shown by elevated hsCRP levels.

It may be that the systemic inflammation we observed is an important factor in secondary outcome in these men. Indeed, it is known that stroke patients with a high inflammatory profile have a poor outcome (31–33), possibly by time-dependent recruitment and activation of inflammatory cells at the site of the injury. Indeed, testosterone is known to affect the immune system (34). Interestingly, a high prevalence of venous thrombo-embolic complications has been documented in Klinefelter Syndrome (35), characterized by genetically low testosterone and often accompanied by abdominal adiposity and metabolic syndrome. It could well be that the relation between low testosterone and secondary outcome is mediated by changes in vascular hemostasis and thrombosis. Testosterone is known to affect platelets (36), but an effect on thrombosis has not been found (37).

From a disease progression point of view, testosterone could be more important in the development and progression of atherosclerotic disease in earlier stage of the disease, instead of in older patients with advanced atherosclerosis as we have studied here. Animal studies indeed point toward a beneficial effect of testosterone during the development of the atherosclerotic plaque (7, 8), but longitudinal studies in humans are inconclusive as excellently reviewed elsewhere (38). The premature interruption of the Testosterone in Older Men With Mobility Limitations (TOM) study raised serious concerns on the potential increased risk of CVD upon testosterone supplementation (39).

### Limitations of the Study

Testosterone levels were available in 203 men and our study may not be sufficiently powered to assess associations of each individual gene with plasma testosterone levels. However, a predefined testosterone responsive list of genes also did not reveal any association.

In literature, we searched for candidate genes that are influenced by the androgen receptor. Expression levels of these genes were associated with plaque androgen receptor expression and testosterone levels. Publicly available data was scarce and limited to expression levels obtained in endothelial progenitor cells. Although the endothelial cell is considered to be a central player in the effects of testosterone on the vessel wall, we cannot rule out the possibility that co-expression of relevant genes can be found in other cell clusters present in atherosclerotic lesions.

In our study, the expression of the androgen receptor does show a correlation with downstream gene expression, hinting toward an association between the testosterone pathway and down-stream gene expression, but was independent of plasma testosterone levels. As dihydrotestosterone (DHT) is the most active androgen, for future studies, it might be interesting to assess the association between different DHT levels and the plaque transcriptome, instead of total testosterone alone. Unfortunately, we did not measure DHT in this study.

Lastly, testosterone levels depend on aromatase-activity, as testosterone is converted to estradiol (1). This aromatization is dependent on white adipose tissue, and thereby obese subjects often have lower testosterone levels. Thus, after conversion to estradiol, testosterone can theoretically also have an effect on plaque expression indirectly via estrogen-receptors, a link that needs to be studied further.

## Conclusion

In men with severe atherosclerotic disease the androgen receptor is highly expressed in plaque tissue. However, plasma testosterone levels were neither associated with gene expression profiles nor with gene regulatory networks or with pre-selected testosterone sensitive genes in late-stage atherosclerotic plaques. The effect of testosterone on gene expression of the late-stage atherosclerotic plaque appears limited, suggesting that alternative mechanisms explain its effect on clinical outcomes.

## Data Availability Statement

The datasets generated for this study can be found in online repositories. The names of the repository/repositories and accession number(s) can be found in the article/Supplementary Material.

## Ethics Statement

The studies involving human participants were reviewed and approved by University Medical Center Utrecht Medical Research Ethical Committee. The patients/participants provided their written informed consent to participate in this study.

## Author Contributions

FG undertook the writing of the paper and initiated the analysis plan. EDB guided the writing of the paper and analyzed the data. AB analyzed the data and made substantial improvements to the paper. NT, FW, RH and GP made substantial improvements to the paper. NO-M, GP and HR supervised the study and made substantial improvements to the paper. All authors have read and given final approval of the submitted manuscript.

## Conflict of Interest

The authors declare that the research was conducted in the absence of any commercial or financial relationships that could be construed as a potential conflict of interest.
