# Supplementary material for: Plasma Testosterone Levels and Atherosclerotic Plaque Gene Expression in Men With Advanced Atherosclerosis
Source: Front Cardiovasc Med. 2021 Jun 14;8:693351. doi: 10.3389/fcvm.2021.693351 (PMC8236711; doi:10.3389/fcvm.2021.693351)
Supplement: Supplementary file 1 [file Data_Sheet_1.PDF]

Supplementary Information

Supplementary Figures

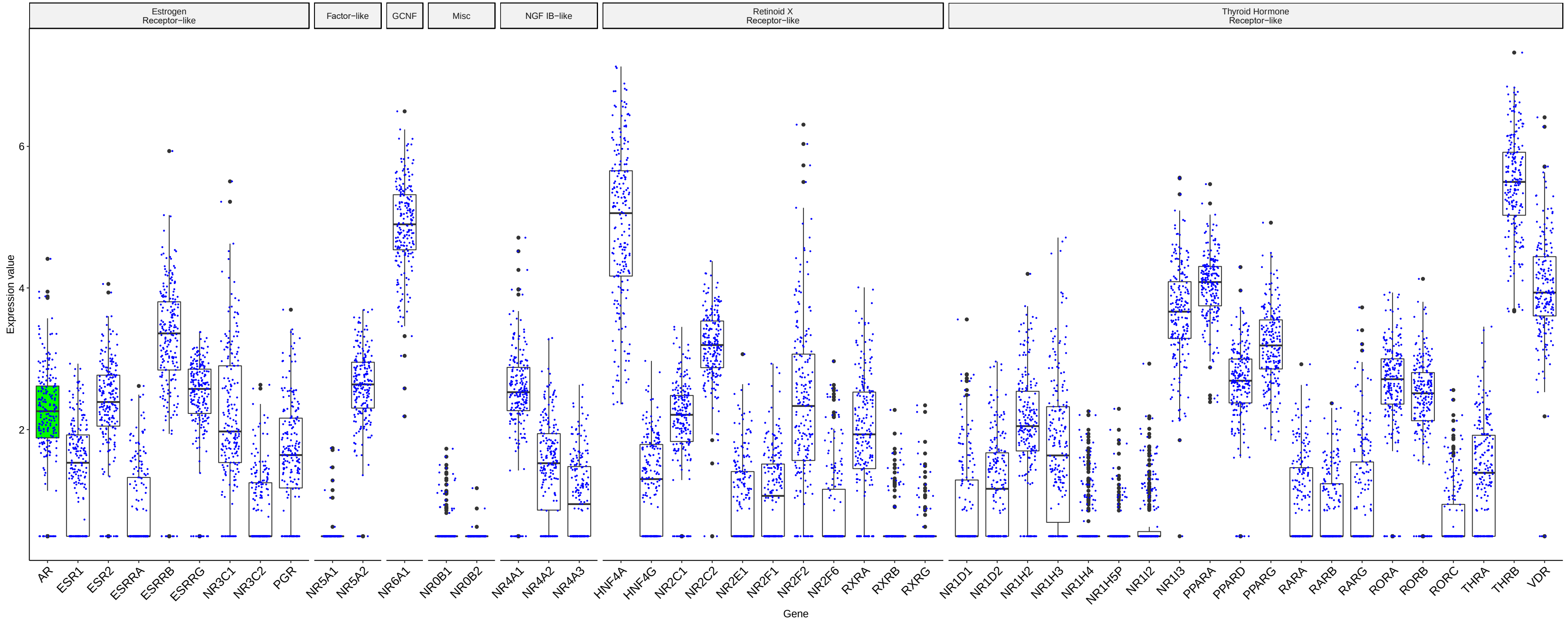

Figure S1. Expression levels of nuclear receptors in comparison with the androgen receptor (AR) gene in green for 203 carotid samples from the AtheroExpress endarterectomy biobank. Genes have been grouped based on their subfamily.

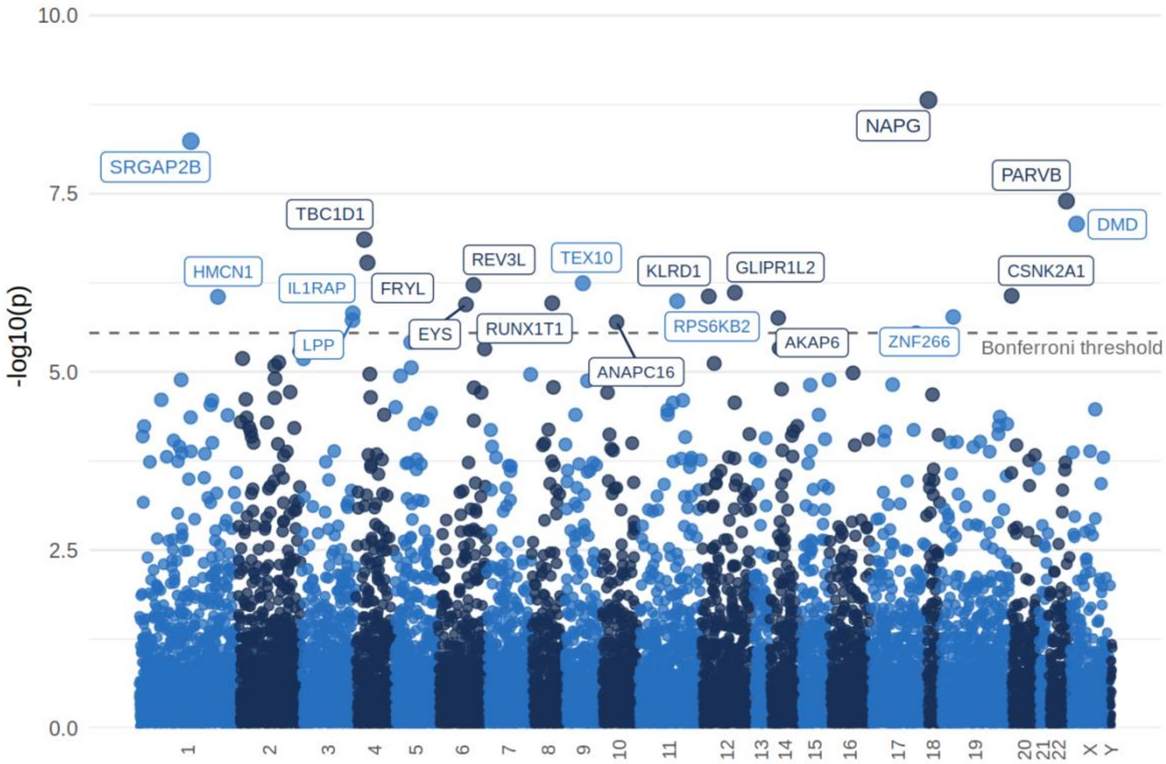

Figure S2. Manhattan plot of gene expression association with androgen receptor (AR) gene expression. Genes with significant p-value after Bonferroni correction for multiple testing are labelled in the plot.

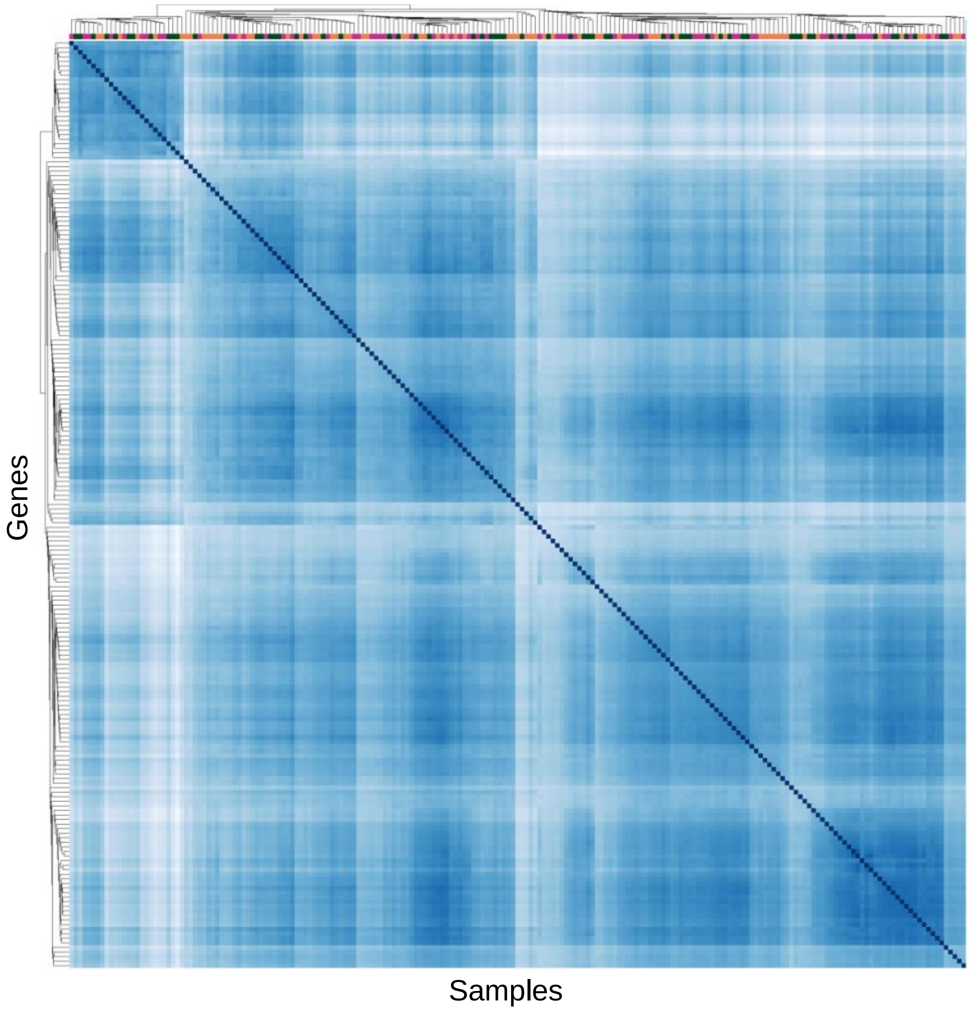

Figure S3. Hierarchical clustering of samples based on expression of testosterone-sensitive genes associated with either the androgen receptor (AR) gene expression or based on previous literature.

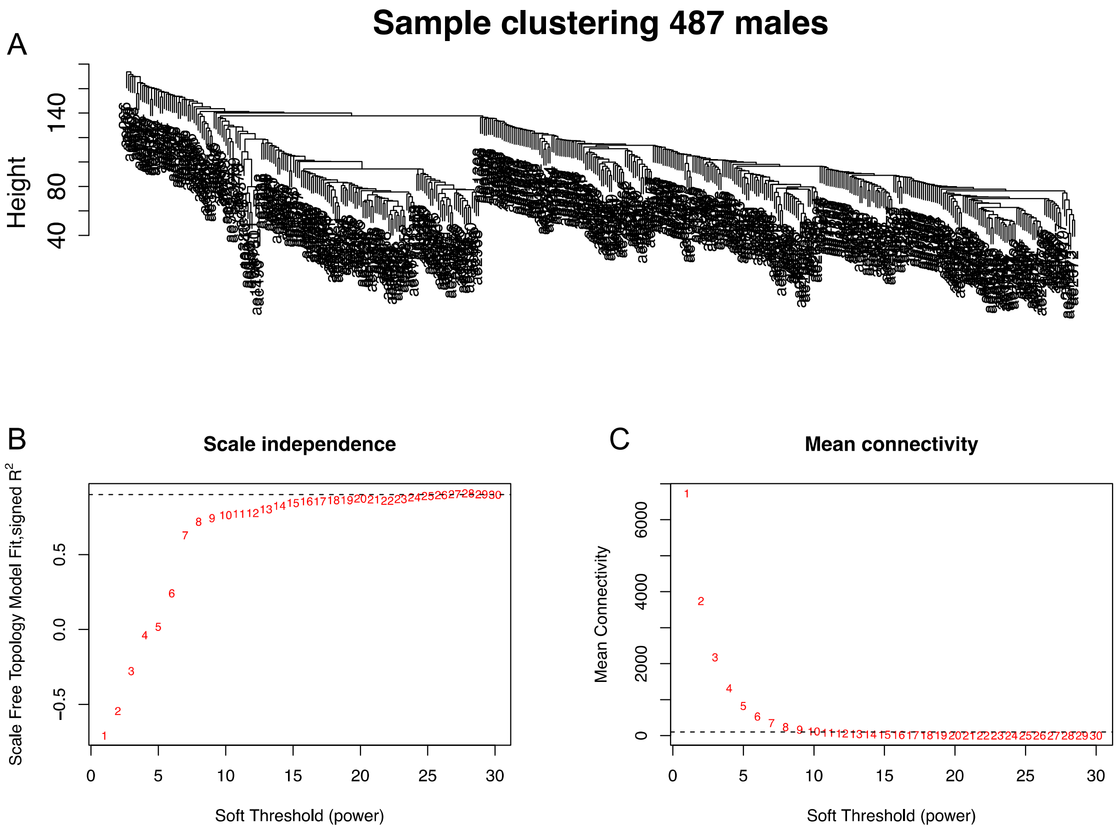

Figure S4. Gene expression module generation in 487 males. A) Hierarchical clustering of samples included in the study for generation of co-expression modules. No samples were dimed outliers. B) Scale free topology Model fit for different soft-thresholds, the dashed line represents the selection cut-off of 0.8 R^2. C) Mean connectivity for the genes in the networks, cut-off for selection of relevant soft-threshold was set at mean connectivity = 100 (dashed line).

Supplementary Tables

Table S1. Genes significantly correlated with Androgen Receptor (AR) gene expression in males undergoing carotid endarterectomy. The genes presented remained significant after Bonferroni correction for multiple testing.

Gene symbol	Correlation coefficient	p-value	Test type	Adjusted p-value	Target of the AR (Transcription factor)
NAPG	0.43	1.54E-09	two.sided	2.87E-05	No
SRGAP2B	0.41	5.80E-09	two.sided	5.41E-05	No
PARVB	-0.39	4.01E-08	two.sided	2.49E-04	No
DMD	0.38	8.43E-08	two.sided	3.93E-04	Yes
TBC1D1	0.37	1.40E-07	two.sided	5.21E-04	Yes
FRYL	0.37	2.95E-07	two.sided	9.16E-04	No
CSNK2A1	-0.35	8.60E-07	two.sided	1.38E-03	Yes
GLIPR1L2	0.35	7.75E-07	two.sided	1.38E-03	Yes
HMCN1	0.35	8.87E-07	two.sided	1.38E-03	Yes
KLRD1	0.35	8.74E-07	two.sided	1.38E-03	No
REV3L	0.35	6.05E-07	two.sided	1.38E-03	No
TEX10	0.36	5.71E-07	two.sided	1.38E-03	No
EYS	0.35	1.14E-06	two.sided	1.41E-03	Yes
RPS6KB2	-0.35	1.02E-06	two.sided	1.41E-03	Yes
RUNX1T1	0.35	1.09E-06	two.sided	1.41E-03	No
IL1RAP	0.34	1.50E-06	two.sided	1.75E-03	Yes
AKAP6	0.34	1.75E-06	two.sided	1.82E-03	Yes
ZNF266	-0.34	1.70E-06	two.sided	1.82E-03	Yes
LPP	0.34	1.88E-06	two.sided	1.85E-03	Yes
ANAPC16	-0.34	2.01E-06	two.sided	1.87E-03	Yes

Table S2. Top 15 genes nominally correlated with Testosterone levels after Bonferroni correction for multiple testing in men undergoing carotid endarterectomy.

Gene symbol	Correlation coefficient	*p*-value	Test type	Adjusted *p*-value
DELE1	0.26	1.95E-04	two.sided	0.42
MEIS3	0.26	1.77E-04	two.sided	0.42
NFRKB	0.26	2.01E-04	two.sided	0.42
NKIRAS2	0.27	1.27E-04	two.sided	0.42
RPS6KB2	-0.26	1.59E-04	two.sided	0.42
SLC46A1	-0.27	8.64E-05	two.sided	0.42
TAOK3	0.26	1.59E-04	two.sided	0.42
WDR92	-0.26	2.04E-04	two.sided	0.42
ZFYVE9	0.26	1.41E-04	two.sided	0.42
CENPS	-0.25	2.64E-04	two.sided	0.49
IFNG	-0.24	6.68E-04	two.sided	0.87
KCNIP2	-0.24	5.67E-04	two.sided	0.87
NLRP2	0.24	7.02E-04	two.sided	0.87
OR5D16	0.24	7.11E-04	two.sided	0.87
PADI4	-0.23	7.45E-04	two.sided	0.87
POLR2H	0.24	6.95E-04	two.sided	0.87
NKX3-2	0.23	8.56E-04	two.sided	0.94
